# Effects of Yogic Practices Synchronized With Bandha and Kumbhaka on Biological and Psychological Factors of Aging in COVID-19-Recovered Patients: A Randomized Controlled Trial

**DOI:** 10.7759/cureus.71884

**Published:** 2024-10-19

**Authors:** Anuj Kumari, Ajay Pal, Rima Dada

**Affiliations:** 1 Yoga Sciences, Central University of Haryana, Mahendergarh, IND; 2 Anatomy, Laboratory for Molecular Reproduction and Genetics, All India Institute of Medical Sciences, New Delhi, New Delhi, IND

**Keywords:** aging, anemia, antioxidants, bandha, health span, kumbhaka, longevity, mental stress, oxidative stress, yoga

## Abstract

Background and objectives

Accelerated biological aging and age-associated diseases are strong risk factors for mortality and morbidity. Oxidative stress (OS) and anemia are possible pathophysiological causes of the various organ dysfunctions observed during COVID-19, decreasing health and life span. Ancient Yogic science seems to optimize all dimensions of human existence. As mentioned in ancient Yogic scriptures and documented in various studies, Yoga has been found to control accelerated biological aging and associated diseases. The study's objective was to authenticate and look into the effect of Yogic practices specifically synchronized with Kumbhaka and Bandha on markers of accelerated aging.

Methods

This randomized controlled trial was carried out in Mahendergarh city of Haryana on COVID-19-recovered adults aged between 30 and 60 years; 126 adults were randomized into two groups from Mahendergarh city: a control group (CG), 61 adults, and the experimental group (EG), 65 adults. During the final analysis, 56 adults in the experimental group received Yogic intervention for 120 days, and 61 adults remained the same in the control group during the intervention period. Consenting participants were randomized using computer-generated block randomization. The Yogic intervention was done 60 minutes/day five days a week for six months. Both groups' laboratory tests were carried out, which included malondialdehyde (MDA) level, total antioxidant capacity (TAC), glutathione (GSH) levels, hemoglobin (Hgb) level, body mass index (BMI), mental stress (perceived stress), and quality of life (QOL), which were estimated before and after the Yogic intervention.

Results

Yoga practice for 120 days (three mandals) in the experimental group has significantly reduced MDA level (p = 0.03) and perceived stress level (Perceived Stress Scale {PSS}) (p = 0.047), and BMI decreased in the Yoga group from 24.2 ± 4.8 to 23.6 ± 4.8, but no significant difference was observed in the values of BMI (p = 0.54). Improved antioxidant levels such as GSH level (p = 0.02), serum ferric-reducing antioxidant power (FRAP)/TAC activity (p = 0.04), and Hgb level (p = 0.02) were reported; with this, improved quality of life, World Health Organization Quality of Life (WHOQOL) Physical (p = 0.03), WHOQOL Psychological (p = 0.02), WHOQOL Social (p = 0.04), and WHOQOL Environment (p = 0.006), has been observed in the experimental group, whereas in the control group, we observed no significant difference in MDA level (p = 0.38), GSH level (p = 0.97), TAC level (p = 0.96), Hgb level (p = 1), BMI (p = 0.85), PSS (p = 0.83), and quality of life, WHOQOL Physical (p = 0.37), WHOQOL Psychological (p = 0.88), WHOQOL Social (p = 0.96), and WHOQOL Environment (p = 0.32).

Conclusion

These findings suggest that Yoga synchronized with Kumbhaka and Bandha may be a useful strategy for lowering oxidative stress and mental stress and improving antioxidant defense, hemoglobin level, and overall quality of life in COVID-19-recovered people, which might help reverse the biological decline of the human body and mind. The results of this study show that Yoga may break the link between old age and ill health. Hence, Yoga (with Bandha and Kumbhaka)* *may be* *the most reproducible way to extend the life span of humans, as mentioned in ancient Yogic scriptures.

## Introduction

Swami Vivekananda said, "As long as the body lives, there must be strength in the body, mind, and hand." Similarly, as mentioned in ancient Yogic texts, Atharva Veda 19:67, we see, live, know, grow, and thrive for a hundred autumns. However, the world's population continues to grow at an unprecedented rate; older individuals are rapidly increasing. According to the Census 2011 in India, older people aged ≥60 are 8.6% of the total population and are estimated to be about 19% or above by 2050 [1]. Aging is the decline of homeostasis over time, resulting in a physiological decline or imbalance of all the monitoring systems of the body, leading to unhealthy and shorter life. Humans are living longer but not healthier, and their health is steadily declining, which is a major challenge. The older population accounts for the largest share of the spectrum of chronic disorders [2]. Disability and disease are associated with an aging population, because increased developments in medical technology have extended the life span but not the health span [3]. The prevalence of age-related diseases (cardiovascular diseases, lung diseases, stroke, malignant diseases, osteoporosis, musculoskeletal disorders, and metabolic syndrome) is very high [4,5]. Cardiopulmonary and metabolic disorders are associated with the aging world [6]. The increased oxidative stress (OS) is a possible pathophysiological cause of the various organ dysfunctions observed during the post-COVID-19 period [7]. Thus, post-COVID-19 symptoms may remain throughout life and weaken the immune system through long-term complications, increased oxidative stress, DNA damage, and disturbed epigenetic clock decreasing health and life span [8]. Due to increased biological aging, spending on anti-aging products has continuously risen. Therefore, further research is required to find a modest, suitable, and cost-effective alternative to slow the aging process and related illnesses and to improve overall health span.

According to a government survey, anemia increased from 53% to 57% in females from 2016 to 2019, and it was the fifth highest worldwide [9]. The National Family Health Survey (NFHS) reported that anemia among females increased from 52% to 53% in 15 years [9,10]. The World Congress of Gerontology, Vancouver, Canada (2001), held the symposium "Anemia in the Elderly"; one of the chief themes was that the occurrence of anemia rises with time of life [11]. The World Health Organization standards for anemia are that hemoglobin (Hgb) below 12 g/dL in females and below 13 g/dL in males are considered anemic people. A third of the world's population suffers from anemia, which impairs neurological development, increases illness and mortality, and reduces worker efficiency [12]. Anemia causes a chronic decrease in the ability to carry oxygen, which reduces intellectual, physical, and functional abilities [13]. It also lowers economic production and increases the risk of infection because of its effects on immunity, morbidity, and death. The high occurrence of low hemoglobin and hematocrit, specifically in rural areas, is due to a lack of nutrition education and abnormal diet and lifestyle. Several factors, for example, blood loss or long-lasting disease, may be responsible for anemia [14]. Sometimes, the cause is still a mystery. Therefore, it would appear essential to diagnose and treat anemia in the elderly population effectively.

The goal of the current investigation was to know the impact of specific Yogic intervention on oxidative stress, Hgb level, stress, and quality of life (QOL) in aging adults; to achieve the goals of "Anemia Mukt Bharat"; and to prevent diseases with aging and to improve overall health span [15]. Today, several recent research findings point out that an appropriate lifestyle with physical activity, anxiety reduction, and proper nutrition and longevity genes promotes slow and disease-free aging [16,17]. Therapies that attenuate psychological and oxidative stress have proved essential for a healthy life and disease-free slow aging. Aurobindo states that Yoga is a mind-body-spirit discipline that enhances psychosomatic health and well-being and unites the individual soul with the universal soul [18]. Similarly, Iyengar and Menuhin explain that physical, mental, emotional, and spiritual well-being are the four aspects of human existence that Yoga aims to optimize [19]. The ancient ethics and practices of Yoga enhance immunity, delay aging, prevent illness, and promote health. The Hatha Yoga incorporates Shatkarmas, Asanas, Mudras, Bandha, Pranayama, Dharana, and Dhyana. Yoga proved effective in hypertension, diabetes, and other chronic disorders [20-22]. Thus, there is a need for additional investigation on reverse aging to assess the effect and mechanism of Yoga on health and the aging process.

Various agents or therapies (antioxidants, telomeres, drugs, stem cells, nutrition, and physical exercise) have been investigated to ascertain their impact on extended health and life span. Nevertheless, no single treatment is completely safe, efficient, and effective, due to the unknown repercussions and side effects of all the therapies on the human body [23]. Additionally, these treatments have demonstrated negative consequences such as nephrotoxicity, platelet depletion, erratic metabolism, altered blood pressure (BP) or intracranial pressure, increased fat and sugar levels, and even malignancy [24-29]. Various research has attested to the causal beneficial effects of mind-body treatment on age reversal markers. Still, very little research provides information on the mechanism of mind-body intervention on age reversal markers and references to the old Yogic literature. As mentioned in the ancient Yogic text Svetasvatara Upanishad, verse 12 states that a yogi who carries the fire of Yoga within him will not be impacted by illness, aging, or death. Therefore, further investigation on reverse aging is required to identify a low-risk, appropriate, and affordable alternative to extend life expectancy. The effectiveness and safety of Yogic practices are relatively high. Hence, Yoga may prove a cost-effective substitute that improves health span by removing all sufferings and preventing the harmful effects of other treatments. To enhance health span, prevent illness, boost immunity, heal, and slow aging, there is a need to integrate traditional Yogic concepts with the Western medical system and psychology.

The Yogic practice of Kumbhaka and Bandha is still not widely utilized, and limited studies are available on its efficacy. Very little data is available about the impacts of Kumbhaka and Bandha on hemoglobin levels and oxidative stress. Thus, we investigated if Yogic practice with Kumbhaka and Bandha affects an adult's Hgb level, oxidative stress, mental stress, and quality of life.

## Materials and methods

Subjects

**Figure 1:**
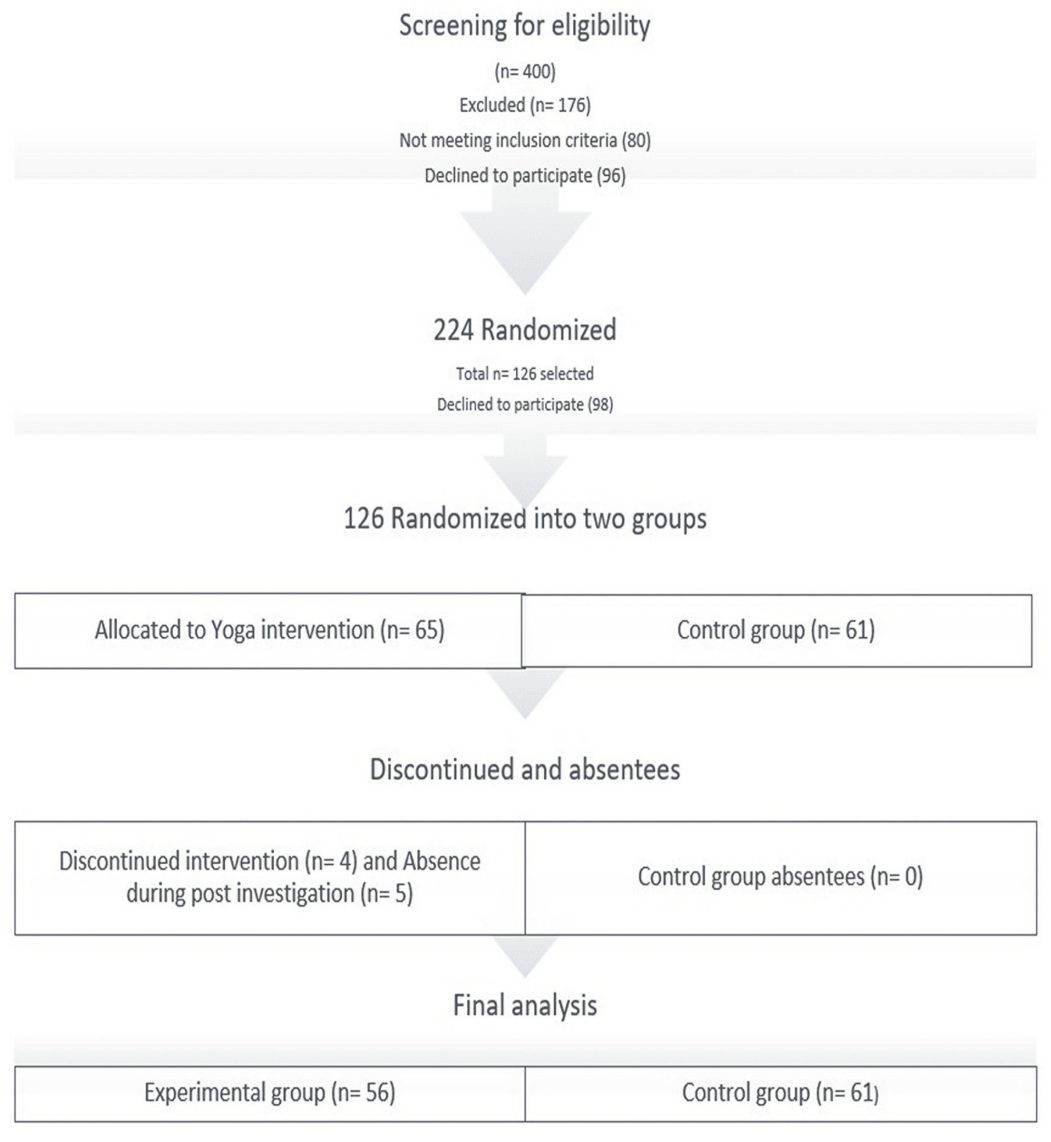
Study participants n: numbers of participants

The pre-post randomized controlled trial was conducted at Mahendergarh city of Haryana with the collaboration of civil hospitals in Mahendergarh and Narnaul. For six months from July 2022 to December 2022, Yogic intervention was done 60 minutes/day five days a week. The study samples consisted of COVID-19-recovered middle-aged adults from different colonies of Mahendergarh city aged between 30 and 60 years (Figure 1). After screening 400 people, 126 samples were randomized into two groups using computer-generated block randomization at the Central University of Haryana. The control group consisted of 61 people, and the experimental group consisted of 65 people; four individuals discontinued, and five were absent during post-investigation. Hence, during the final analysis, 56 people (Figure 1) were in the experimental group and received Yogic intervention for 120 days, and 61 people (Figure 1) remained in the control group. Other noteworthy medical and surgical history details were recorded. Both groups' laboratory tests were carried out, at baseline and after 120 days of Yogic intervention. The Yogic intervention includes prayer, Asana, Pranayama, and Mudra all synchronized with Kumbhaka, Bandha, and awareness.

Inclusion Criteria

The inclusion criteria included COVID-19-recovered people from Mahendergarh city; the participants should not be involved in any other exercise and be between 30 and 60 years old and both genders.

Exclusion Criteria

The inclusion criteria included pregnant female participants with any medical health issues or chronic diseases such as mental retardation and cancer, COVID-19-positive patients, and patients with age below 30 years.

Ethical consideration

The bilingual informed consent form was provided to the participants, and this study is ethically approved by the institutional human ethics committee of the Central University of Haryana (approval number: CUH/IHEC/2022/02) to defend the safety, dignity, rights, and well-being of the participants as per the national ethical guidelines. The clinical trial registry of the study was CTRI/2022/05/042896 (registered on 30/05/2022), Trial Registered Prospectively of India.

Study design

A randomized controlled study was conducted on COVID-19-recovered people (n = 126) between 30 and 60 years old.

Variable

Dependent Variable

Oxidative stress: The disturbed free radicals and antioxidant levels in the body lead to DNA damage and cellular death, accelerating the aging process [30]: (a) Glutathione (GSH) is an antioxidant synthesized in the cell that protects oxidative DNA damage and sustains redox homeostasis of the cell by removing various reactive species such as superoxide and hydrogen peroxide and preventing oxidative damage [31], (b) malondialdehyde (MDA) indicates high oxidative stress and lipid (polyunsaturated fatty acids) peroxidation [32] in the body's cells, and (c) ferric-reducing antioxidant power/total antioxidant capacity (FRAP/TAC) is used to determine the total antioxidant level or total antioxidant activity in the plasma by reducing the ferric ion to the ferrous ion [33].

Hemoglobin: Hemoglobin is a red protein responsible for transporting oxygen. The occurrence of anemia in older people is 4-6 times more than that assumed clinically and increases with age [34]. Hemoglobin is measured by using a TrueHb Hemoglobinometer. Neogi et al. (2016) reported that TrueHb performs better with venous than capillary blood samples. TrueHb had sensitivity and specificity values of 82% and 78%, respectively, and was well correlated with the autoanalyzer (r = 0.77) [35].

BMI: Body mass index (BMI) is measured around 10:00 am without sleepers and light clothes. A stadiometer is used to measure height to the nearest 0.5 cm. The digital weighing machine is used to measure weight to the nearest 100 g. BMI is calculated by dividing weight in kilograms by the height in square meters (BMI = kg/m^2^).

Excellence in life (World Health Organization Quality of Life Brief Version {WHOQOL-BREF}): The questionnaire by the WHO for excellence of life is a frequently used instrument to assess the quality of life (physical, psychological, social, and environmental domains) in both healthy and ill populations [36]. Cronbach's alpha was 0.85, 0.83, 0.62, and 0.81, respectively, for the physical, psychological, social, and environmental domains and 0.92 for the total scale. The level of internal consistency was acceptable.

Stress (Perceived Stress Scale-10 {PSS-10}): The Perceived Stress Scale (PSS) is used to measure somatic, emotional, mental, social, and behavioral perceived stress. Stress is directly linked to the biological aging process [37]. Internal consistency reliability for the PSS-10 total scores was adequate for the full sample (α = 0.82) [38].

Independent Variable

Yogic intervention: The experimental group will receive the Yogic intervention for 120 days (three mandals; one mandal = 40 days), 60 minutes/day five days a week. The control group (CG) will not receive Yogic intervention during the intervention period.

In the initiation of Yogic intervention with prayer, the Gayatri Maha mantra (Rigveda 3/62/10) was recited for three rounds. Prayer enhances positive emotions such as satisfaction, peace, and attention and improves mood stability. Neurophysiology is associated with religious mantra chanting or prayer. It induces calmness and nurtures relaxation [39]. Asanas such as Uttanpadasana, Pawanmuktasana, Bhujangasana, Salabhasana, Vajrasana, Singhasana, Tadasana, and Padhastasana were performed three rounds each for 15 minutes. Asanas improve the anatomical and physiological functions of the body through massage, blood circulation, and oxygen supply to body cells, which induces homeostasis [40]. Pranayama synchronized with specific Bandha and Mudra such as Nadi Shodhan, Bhastrika, Ujjayi, Shitkari, Kapol Sakti Vikasak, and Bhramari was performed three rounds each for 20 minutes. Nadi Shodhan clears both nasal passages and regulates nasal cycles [41]. Bhastrika improves blood and lymph circulation and improves cognitive functioning [42]. Ujjayi and Shitkari activate parasympathetic functioning by improving sleep quality and the oxygenation of the blood, balancing BP, and increasing cardiac output [43]. Kapol Sakti Vikasak improves cardiopulmonary and neurological health. Bhramari removes negative emotions such as stress, anxiety, frustration, and depression by inducing positive emotions such as concentration and patience [42]. Bandha such as Jalandhar and Uddiyan Bandha were performed for three rounds each, and Mudras such as Vipritkarni Mudra and Prana Mudra were performed for three rounds each for 15 minutes. Breath retention improves immune functions and adapts the body to extreme conditions such as acidosis, stimulating dormant cells and vasculature [44,45]. Om recitation for five minutes enhances the secretion of tranquilizing neurohormones such as serotonin, anandamides, enkephalin, and endorphin and improves the structure and function of the brain such as increased gray matter and increased δ-wave and α-wave generation [46]. Shanti mantra for two minutes induces calmness and nurtures relaxation [39]. Moreover, it enhances the stability of cardiac functions [47].

Data extraction method

Laboratory testing of biomarkers such as GSH, MDA, and FRAP through an enzyme-linked immunosorbent assay (ELISA) reader was conducted in the Department of Biochemistry at the Central University of Haryana: erythrocyte glutathione (GSH) method, the ability of the sulfhydryl group of GSH peptide to reduce 5,5'-dithiobis-(2-nitrobenzoic acid) (DTNB); malondialdehyde (MDA) method by Esterbauer and Cheeseman; and ferric-reducing antioxidant power (FRAP) method by Benzie and Strain, antioxidant capacity assay/reductive potential assay/reductive power of antioxidants.

Data analysis

The obtained data was expressed as mean, standard deviation, and p-value. An unpaired independent, as well as dependent, t-test was applied for the determination of statistical significance. Statistical significance was established at p < 0.05. Microsoft® Excel® 2021 MSO (Version 2306 Build 16.0.16529.20164, 64-bit) (Microsoft Corp., Redmond, WA) was used for data analysis.

## Results

The result shows the baseline characteristics (Table 1) of the experimental and control group participants; there were no significant changes observed in the baseline values of MDA (p = 0.74), GSH (p = 0.87), FRAP (p = 0.96), Hgb (p = 0.50), BMI (p = 0.79), WHOQOL Physical (p = 0.90), WHOQOL Psychological (p = 0.78), WHOQOL Social (p = 0.66), WHOQOL Environment (p = 0.86), PSS (p = 0.70) between the control and experimental groups.

**Table 1:** Demographic, biochemical, and psychological pre-data (baseline) GSH, glutathione; MDA, malondialdehyde; FRAP, ferric-reducing antioxidant power; Hgb, hemoglobin; BMI, body mass index; WHOQOL, World Health Organization Quality of Life

Demographic, biochemical, and psychological pre-data	Total samples (N = 126)	Experimental group (EG) (n = 65)	Control group (CG) (n = 61)	P-value
Age	42.95 ± 8.24	42.14 ± 8.14	43.82 ± 8.12	0.253
Gender	49, male; 77, female	25, male; 40, female	24, male; 37, female	-
BMI	24.31 ± 4.77	24.20 ± 4.87	24.43 ± 4.71	0.79
GSH	169.58 ± 63.73	160.93 ± 46.37	162.37 ± 52.13	0.87
MDA	10.96 ± 5.46	10.94 ± 5.23	11.15 ± 5.73	0.74
FRAP	569.21 ± 97.30	568.40 ± 111.65	569.80 ± 80.17	0.96
Hgb	9.83 ± 1.79	10.22 ± 1.70	10.00 ± 1.78	0.50
WHOQOL Physical	50.48 ± 9.97	50.94 ± 11.24	51.15 ± 7.74	0.90
WHOQOL Psychological	49.78 ± 11.45	50.60 ± 12.79	50.01 ± 9.12	0.78
WHOQOL Social	48.56 ± 16.41	50.03 ± 17.74	51.31 ± 14.91	0.66
WHOQOL Environment	49.66 ± 10.78	49.83 ± 11.95	49.49 ± 9.94	0.86
Perceived Stress Scale (PSS)	20.97 ± 6.87	20.74 ± 6.49	21.21 ± 7.78	0.70

After 120 days of intervention, there were significant changes observed between the control and experimental groups (Table 2) in the values of MDA (p = 0.03), GSH (p = 0.02), FRAP (p = 0.003), Hgb (p = 0.001), WHOQOL Physical (p = 0.01), WHOQOL Psychological (p = 0.004), WHOQOL Social (p = 0.05), WHOQOL Environment (p = 0.02), and PSS (p = 0.0001). However, BMI decreased after intervention in the experimental group but did not show a significant difference (p = 0.27).

**Table 2:** Demographic, biochemical, and psychological post-data GSH, glutathione; MDA, malondialdehyde; FRAP, ferric-reducing antioxidant power; Hgb, hemoglobin; BMI, body mass index; WHOQOL, World Health Organization Quality of Life

Demographic, biochemical, and psychological post-data	Total samples (N = 117)	Experimental group (EG) (n = 56)	Control croup (CG) (n = 61)	P-value
Age	43.16 ± 8.23	42.45 ± 8.09	43.82 ± 8.12	0.369
Gender	46, male; 71, female	22, Male; 34, female	24, male; 37, female	-
BMI	24.34 ± 4.88	23.66 ± 4.85	24.58 ± 4.16	0.27
GSH	170.31 ± 37.96	177.62 ± 35.52	162.68 ± 39.22	0.02
MDA	9.87 ± 5.68	8.98 ± 5.20	11.20 ± 6.06	0.03
FRAP	580.31 ± 105.01	606.72 ± 103.43	551.16 ± 99.72	0.003
Hgb	10.44 ± 1.57	10.90 ± 1.23	10.01 ± 1.71	0.001
WHOQOL Physical	52.84 ± 5.30	54.09 ± 4.56	51.70 ± 5.16	0.01
WHOQOL Psychological	52.34 ± 8.23	54.54 ± 4.49	50.31 ± 9.28	0.004
WHOQOL Social	53.15 ± 11.33	55.32 ± 9.68	51.17 ± 11.78	0.04
WHOQOL Environment	52.69 ± 7.30	54.35 ± 5.58	51.17 ± 8.32	0.01
Perceived Stress Scale (PSS)	20.10 ± 2.70	19.14 ± 1.49	20.98 ± 3.02	0.0001

Table 3 shows the characteristics of the Yoga group participants at baseline and after 120 days. A clear significant difference was observed in the values of GSH (p = 0.02), MDA (p = 0.03), FRAP (p = 0.04), Hgb (p = 0.02), WHOQOL Physical (p = 0.03), WHOQOL Psychological (p = 0.02), WHOQOL Social (p = 0.04), WHOQOL Environment (p = 0.01), and PSS (p = 0.05). BMI decreased, but no significant difference was observed in the values of BMI (p =0.54).

**Table 3:** Experimental group pre-post data analysis GSH, glutathione; MDA, malondialdehyde; FRAP, ferric-reducing antioxidant power; Hgb, hemoglobin; BMI, body mass index; WHOQOL, World Health Organization Quality of Life; SD, standard deviation

Yoga group (experimental group)	Baseline, mean ± SD (N = 65)	After 120 days, mean ± SD (N = 56)	P-value
GSH	160.93 ± 46.37	177.62 ± 35.52	0.02
MDA	10.94 ± 5.23	8.98 ± 5.20	0.03
FRAP	568.40 ± 111.65	606.72 ± 103.43	0.04
Hgb	10.22 ± 1.71	10.90 ± 1.23	0.02
BMI	24.20 ± 4.78	23.66 ± 4.85	0.54
WHOQOL Physical	50.94 ± 11.24	54.09 ± 4.57	0.03
WHOQOL Psychological	50.60 ± 12.79	54.54 ± 4.49	0.02
WHOQOL Social	50.03 ± 17.74	55.32 ± 9.68	0.04
WHOQOL Environment	49.83 ± 11.95	54.35 ± 5.58	0.01
Perceived Stress Scale (PSS)	20.74 ± 6.49	19.14 ± 1.49	0.05

Table 4 shows the characteristics of the control group participants at baseline and after 120 days. There was no remarkable change observed in the values of GSH (p = 0.97), MDA (p = 0.38), FRAP (p = 0.96), Hgb (p = 1), BMI (p = 0.85), WHOQOL Physical (p = 0.69), WHOQOL Psychological (p = 0.88), WHOQOL Social (p = 0.96), WHOQOL Environment (p = 0.32), and PSS (p = 0.83).

**Table 4:** Control group pre-post data analysis GSH, glutathione; MDA, malondialdehyde; FRAP, ferric-reducing antioxidant power; Hgb, hemoglobin; BMI, body mass index; WHOQOL, World Health Organization Quality of Life; SD, standard deviation

Control group	Baseline, mean ± SD (N = 61)	After 120 days, mean ± SD (N = 61)	P-value
GSH	162.37 ± 52.13	162.68 ± 39.22	0.97
MDA	11.15 ± 5.73	11.20 ± 6.06	0.38
FRAP	569.80 ± 80.17	551.16 ± 99.72	0.96
Hgb	10.01 ± 1.78	10.01 ± 1.71	1
BMI	24.43 ± 4.81	24.58 ± 4.19	0.85
WHOQOL Physical	51.15 ± 7.74	51.70 ± 5.16	0.69
WHOQOL Psychological	50.01 ± 9.12	50.31 ± 9.28	0.88
WHOQOL Social	51.31 ± 14.91	51.17 ± 11.78	0.96
WHOQOL Environment	49.49 ± 9.94	51.17 ± 8.32	0.32
Perceived Stress Scale (PSS)	21.21 ± 7.78	20.98 ± 3.02	0.83

Yoga practice has reduced oxidative stress, such as erythrocyte MDA level and perceived mental stress in COVID-19-recovered participants, whereas in the control group, oxidative stress was slightly elevated. Improvement in antioxidant capacity has been observed in the Yoga participants such as total antioxidant capacity/FRAP and GSH level. No such changes were observed in the control group. We have also noticed a significant increase in Hgb level and quality of life (physical, psychological, social, and environmental) in the Yoga group. In contrast, no such changes were noticed in the control group.

## Discussion

Yoga has been found effective in lowering oxidative stress (OS) and improving antioxidant status in COVID-19-recovered people. Aging is a continuous decline of cell, tissue, and organ functions [48]. The oxidative stress theory regarding aging states that functional loss with aging is due to the accumulation of highly reactive oxygen and nitrogen species, accelerating the aging process. A negative correlation between antioxidants and age-associated diseases has already been reported [49]. Growing evidence indicates a strong association between oxidative stress, diseases, accelerated aging, and decreased health and life span [50]. Oxidative stress promotes vascular dysfunction and results in impaired nitric oxide production [51], damages macromolecules (lipid, DNA, protein, and carbohydrates), and accelerates aging and numerous age-associated disorders [49]. A decline in the activity of antioxidants such as glutathione contributes to increased oxidative stress [52]. A glutathione antioxidant acts as a primary line of defense against the toxic effects of reactive oxygen species (ROS). Glutathione peroxidase requires GSH as a coenzyme to convert hydrogen peroxide (H_2_O_2_) to water, and this GSH level was observed low in COVID-19-recovered people, a condition that leads to increased oxidative stress [31].

In the control group participants of this study, we could not find any significant improvement in the GSH level (162.37 ± 52.13 to 162.68 ± 39.22) (p = 0.97) and FRAP/TAC (569.80 ± 80.17 to 551.16 ± 99.72) (p = 0.96) (Table 4). However, in the experimental group of this study, the assessment of antioxidant status established a significant rise in GSH level (160.93 ± 46.37 to 177.62 ± 35.52) (p = 0.022) and FRAP/TAC (568.40 ± 111.65 to 606.7 ± 103.4) (p = 0.04) (Table 3). This is comparable to the study reported by Patil et al. [53] and Hegde et al. [54], who found that Yoga recovers antioxidant status.

After the Yogic intervention in the Yoga group, a significant improvement in erythrocyte GSH and serum antioxidant FRAP/TAC levels was observed, and a significant reduction in erythrocyte MDA (10.94 ± 5.23 to 8.98 ± 5.20) (p = 0.03) (Table 3) level was observed, which is comparable to the findings of Tolahunase et al. [55], Innes et al. [56], and Dada et al. [57]. On the other hand, we found an increase in the erythrocyte MDA (11.15 ± 5.73 to 11.20 ± 6.06) (p = 0.38) (Table 4) in the control group. According to Krishna et al., regular Yoga practice (Asana, Pranayama, and meditation) reduces oxidative stress in older adults [58]. Similarly, according to Dhawan et al. (2018), a Yoga intervention for 21 days in people with frequent pregnancy loss resulted in decreased reactive oxygen species (ROS) levels and oxidative stress [59]. It was reported that after a three-month breath-hold practice, the blood acidosis was reduced with no oxidative stress, and lactic acid also showed improved antioxidant activities [60].

Anemia is a prevalent issue throughout the country and is linked with increased age and multiple diseases [9,61]. The lack of nutrition knowledge and malnutrition have been supposed to be some of the confounders responsible for declining Hgb levels found with increased age [62]. The deficiency of nutrients, particularly iron and B complex, in the usual Indian diet is a serious issue and the consumption of iron and multivitamins with dairy products, which results in an overdose or serious side effects. The consumption of dairy products with iron or multivitamins makes it harder to absorb in the body [63]. Furthermore, drug use and addiction to alcohol, tobacco, and tea are still genuine possibilities.

Erythropoietin stimulates spleen contraction and the production of RBC from the bone marrow [64]. An altered or lowered erythropoietic reserve, or decreased marrow responsiveness, causes anemia with increased age. This study especially focuses on Yogic practices with Kumbhaka and Bandha, which create acidosis by increasing carbon dioxide level in the body, and according to Le Châtelier's principle, when conditions are changed to upset a dynamic equilibrium, the equilibrium position adjusts to restore the original equilibrium by a compensatory mechanism. Hence, the body adapts, accommodates, or equilibrates according to body physiology; in response to hypoxia, the body stimulates spleen contraction, and peripheral vasoconstriction starts nitric oxide production and erythropoietin regulation throughout the body, which improves overall Hgb level. Therefore, it can be assumed that the Yogic practice with Kumbhaka and Bandha may be an effective training method, mainly related to improving endurance capacity, the buffering capacity of the body through adaptation and acclimatization, and restabilizing equilibrium in the body of people having a low level of Hgb and other hematological variables, which results in homeostasis throughout the body. According to this study, a significant difference was observed in the mean Hgb levels. This is similar to the numerous trials with hypoxic training, where the samples experienced hypoxia and the results significantly improved hemoglobin [65,66]. According to this study, the experimental group after practicing Yoga with Kumbhaka and Bandha for 120 days had significantly higher concentrations of Hgb.

We found results in reducing BMI from 24.2 ± 4.8 to 23.6 ± 4.8 (Table 3), which is comparable to the work reported by Chauhan et al. [67], which shows that Yoga practice causes decreased BMI from 26.4 ± 2.5 to 25.22 ± 2.4; the results of this study are not significant in contrast to the findings reported by Telles et al. [68].

According to Lavretsky and Newhouse, stress is linked with shorter telomere length, contributes to accelerated aging, and accelerates biological age [69]. In the present study, stress (21.21 ± 7.8 to 20.98 ± 3.02) (p = 0.83) (Table 4) shows no significant change in the control group, whereas reduced stress (20.74 ± 6.5 to 19.14 ± 1.5) (p = 0.05) (Table 3) was observed in the Yoga group after 120 days of intervention, which is similar to the study by Streeter et al., which found greater decreases in anxiety after 12 weeks of Yoga intervention compared to a walking group [70]. Shohani et al. proved that Yoga seems effective in mental abnormalities [71].

This study aims to assess the quality of life in COVID-19-recovered adults after practicing Yoga. In our research, we found no significant differences in terms of quality of life in the control group: WHOQOL Physical (51.15 ± 7.7 to 51.7 ± 5.2) (p = 0.69) (Table 4), WHOQOL Psychological (50.01 ± 9.1 to 50.3 ± 9.3) (p = 0.88) (Table 4), WHOQOL Social (51.3 ± 14.9 to 51.16 ± 11.7) (p = 0.96) (Table 4), and WHOQOL Environment (49.48 ± 9.9 to 51.17 ± 8.3) (p = 0.32) (Table 4). However, the Yoga group's attained outcomes positively confirmed the excellence of life in the physical (50.93 ± 11.24 to 54.09 ± 4.6) (p = 0.03) (Table 3), psychological (50.60 ± 12.8 to 54.54 ± 4.5) (p = 0.02) (Table 3), social (50.03 ± 17.7 to 55.32 ± 9.7) (p = 0.04) (Table 3), and environmental (49.8 ± 11.9 to 54.35 ± 5.6) (p = 0.01) (Table 3) spheres. Studies that used the WHOQOL-BREF questionnaire produced comparable results, thus indicating the high quality of life in post-COVID-19 individuals during aging. This shows an association between practicing Yoga and the quality of life. The results are consistent with the research conducted before by Piekorz et al. [72].

Kumbhaka with Yogic practice can initiate powerful parasympathetic and sympathetic reflexes, resulting in peripheral vasoconstriction and well-maintained flow to the brain and heart. It has been observed that when Bahir Kumbhaka (external breath-hold) with Maha Bandha was practiced after the rapid breathing of Bhastrika Pranayama, oxygen saturation decreased up to 54%. Once oxygenation goes very low and then high and again returns to its normal value, this going up and down from very high creates hypoxia even though they have enough oxygen [73-75]. Thousands of years before, our sages practiced Kumbhaka extensively to hibernate for many days. Yogic breathing (synchronized with Kumbhaka and Bandha) is to awaken the dormant energy and prana. It has been noticed that Yogic breathing establishes homeostasis throughout the body. Yogic breathing with Bandha and Kumbhaka trains or prepares all organs to work efficiently even in any extreme hypercapnic and anemic conditions, which maintains a balanced state throughout the body and enhances the health span of an individual through maintaining high intrathoracic and intra-abdominal pressures and tones spinal nerves plexuses, neurons, and all other cells of the body. It is noted in the Yoga module of the present study that we have incorporated 15 minutes for Asanas per day synchronized with Kumbhaka, 35 minutes for Pranayama with Bandha and Kumbhaka, five minutes for relaxation (slow deep controlled breath), and five minutes for prayer with "Om" chanting, and mantras were given.

Hence, we presume that Asana and Pranayama (synchronized with Kumbhaka and Bandha) probably reduced oxidative stress and improved antioxidant status in the Yoga practitioners of the present study. To the best of our knowledge, this is the first study reporting on the effect of Yoga especially Kumbhaka and Bandha on oxidative stress and antioxidant defense in COVID-19-recovered people aged between 30 and 60 years in south Haryana. This study can be replicated by evaluating more specific variables such as erythropoietin and other hematological variables as likely factors affecting aerobic capacity, endurance performance, and aging markers such as telomere length in aging people. Anemia and oxidative stress are unsolvable issues that call for creative solutions.

**Figure 2:**
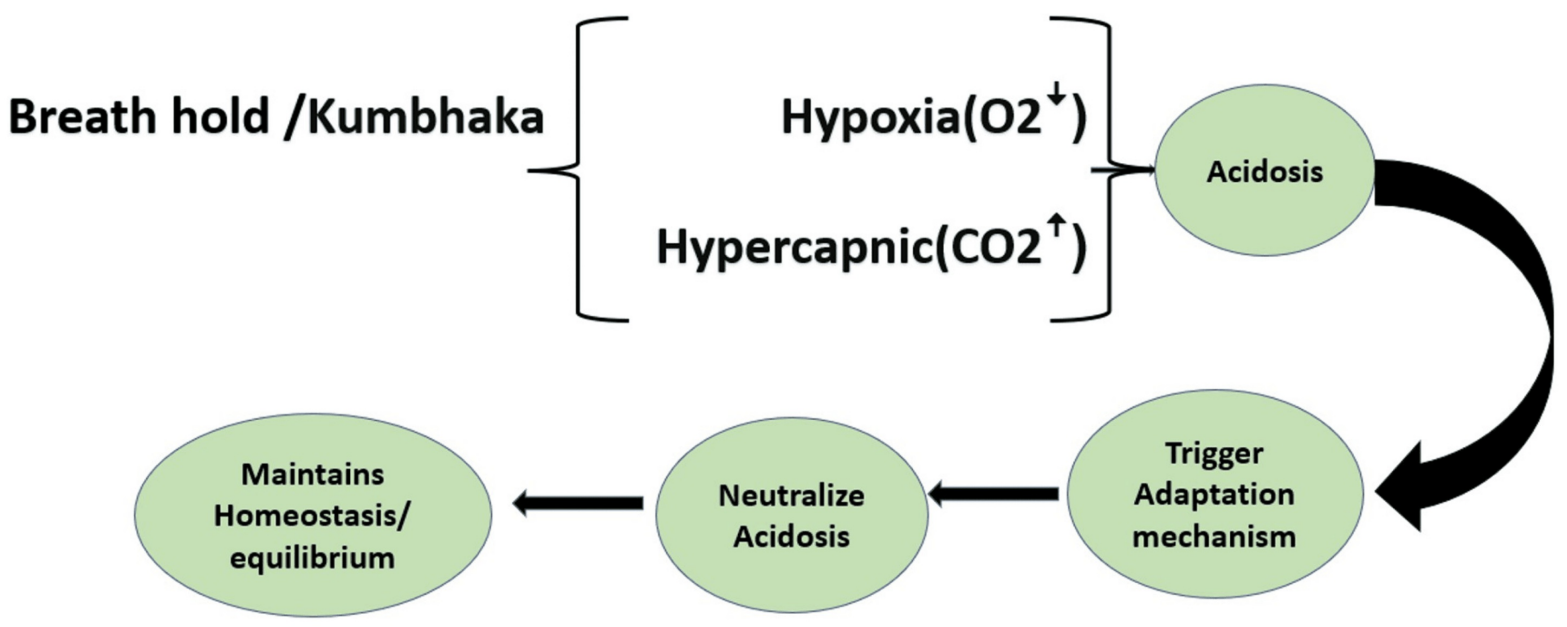
Physiology of Kumbhaka and Bandha stimulates hemoglobin (Hgb) maturation and production

In Figure 2, Kumbhaka and Bandha establish equilibrium according to Le Châtelier's principle on the human body, by stimulating hemoglobin maturation and production through physiological adaptation/buffering, peripheral vasoconstriction, nitric oxide, and erythropoietin production and stimulating spleen contraction and the number of mitochondria, which as a result increases the brain's regenerative capacity.

Limitations

The limitations of this study were samples, which were taken only from COVID-19-recovered people from urban areas, aged between 30 and 60. The samples selected for the study were nonresidential. Therefore, variations in their living conditions, lifestyle, diet, etc., were recognized as a limitation of the study. The experimental group was more aware of diet and lifestyle patterns. This study should be conducted on a larger population and a wide area to generalize its outcomes. Variables were indirectly related to accelerated aging and health span, which should be more specific.

## Conclusions

In contemporary coinage, an effort to integrate the Western medical system and psychology and Yogic practices for well-being, disease inhibition, enhancing immunity, healing, and reverse aging is needed. In Yogic texts, Kumbhaka has greater importance than hundreds of Asanas, Pranayama, and Mudras. Bandha and Kumbhaka at the physical level remove locks simultaneously on mental and pranic levels, affecting and balancing all panchkoshas. The outcomes of this investigation imply that Yoga can be a useful lifestyle tactic for COVID-19-recovered people to lower oxidative stress and strengthen their antioxidant defenses and improve hemoglobin level and quality of life to improve health and lifespan.

According to this study, Yogic practice improves antioxidant defense and quality of life by reducing oxidative damage and stress in the mind and body of COVID-19-recovered individuals, which are the primary causes of the biological decline of the human body and mind. There is an anti-aging possibility, but it has to come from within. Further, it is essential to replicate this type of study at a large level to generalize the outcomes.

## Figures and Tables

**Figure 3:**
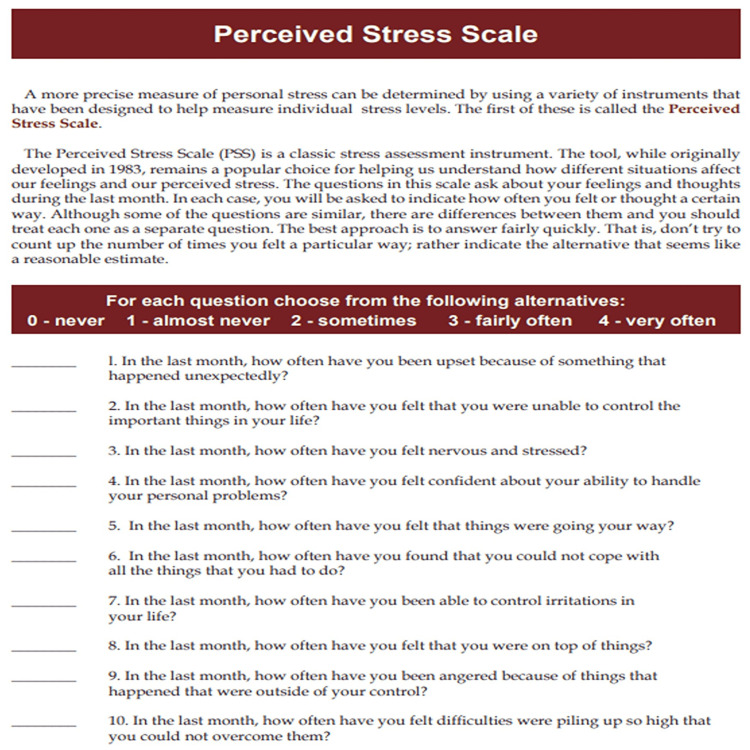
Perceived Stress Scale (PSS)

**Figure 4:**
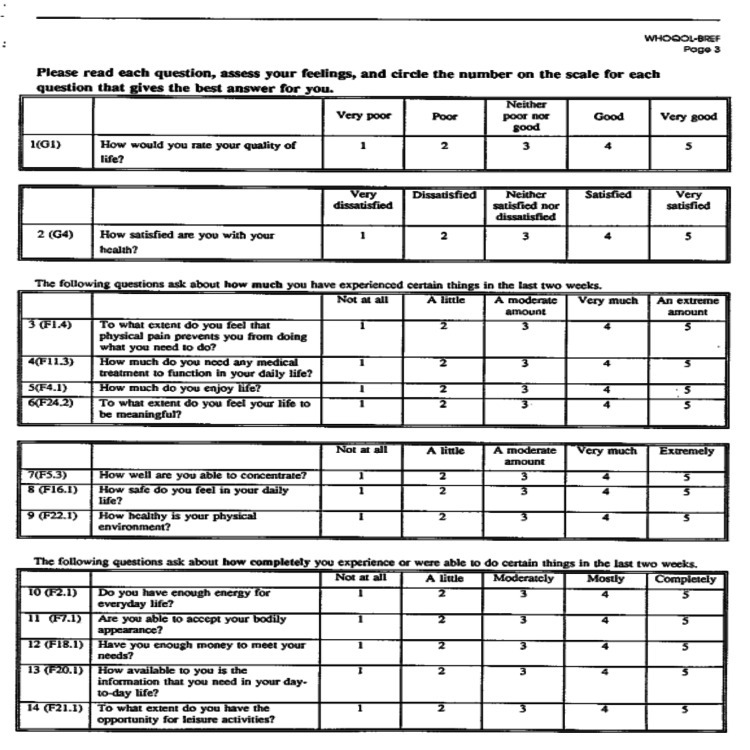
WHOQOL-BREF WHOQOL-BREF: World Health Organization Quality of Life Brief Version

**Figure 5:**
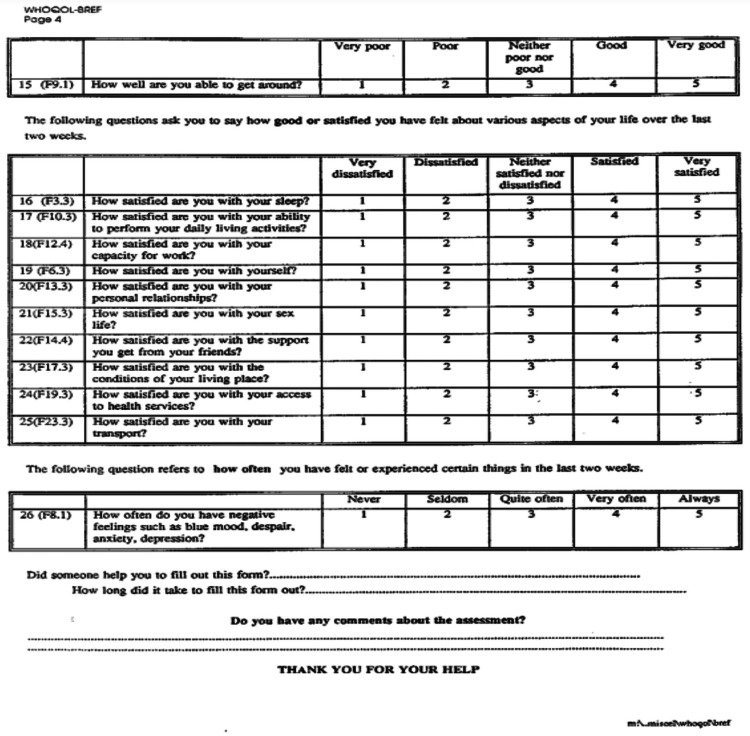
WHOQOL-BREF WHOQOL-BREF: World Health Organization Quality of Life Brief Version

## Appendices

Figure 3 shows the Perceived Stress Scale (PSS) questionnaire, and Figure 4 and Figure 5 show the WHOQOL-BREF questionnaire.
